# Geographical challenges and inequity of healthcare access for high-risk paediatric heart disease

**DOI:** 10.1186/s12939-023-02040-z

**Published:** 2023-11-01

**Authors:** Benjamin C. Auld, Bridget Abell, Prem S. Venugopal, Steven McPhail

**Affiliations:** 1 Queensland Paediatric Cardiac Service, Queensland Children’s Hospital, 501 Stanley St, South Brisbane, QLD 4101 Australia; 2 Australian Centre for Health Services Innovation and Centre for Healthcare Transformation, School of Public Health and Social Work, Faculty of Health, Queensland University of Technology (QUT), Brisbane, Qld 4059 Australia

**Keywords:** Congenital heart disease, Primary care, Hospitals, Geographical information system, Healthcare systems

## Abstract

**Background:**

Geographical context is an important consideration for health system design to promote equality in access to care for patients with childhood heart disease (CHD), particularly those living in regional, rural, and remote areas. To help inform future policy and practice recommendations, this study aimed to (i) describe the geographic distribution of high-risk CHD patients accessing an Australian state-wide specialist service and (ii) estimate travel time for accessing healthcare via general practitioners (primary), nearest paediatric centre (secondary) and specialist paediatric cardiac centre (tertiary).

**Methods:**

Participants included a cohort of children (0–18 year) who accessed state-wide specialist CHD services over a 3-year period (2019–2021) in Queensland, Australia. Locations for patient residence, general practitioner, closest paediatric centre and tertiary cardiac centre were mapped using geographical information system (GIS) software (ArcGIS Online). Travel distance and times were estimated using a Google Maps Application Programming Interface (API).

**Results:**

1019 patients (median age 3.8 years) had cardiac intervention and were included in the sample. Of this cohort, 30.2% lived outside the heavily urbanised South East Queensland (SEQ) area where the tertiary centre is located. These patients travel substantially further and longer to access tertiary level care (but not secondary or primary level care) compared to those in SEQ. Median distance for patients residing outside SEQ to access tertiary care was 953 km with a travel time of 10 h 43 min. This compares to 5.5 km to the general practitioner and 20.6 km to a paediatric service (8.9 and 54 min respectively).

**Conclusion:**

This geographical mapping of CHD services has demonstrated a key challenge inherent in providing specialist cardiac care to children in a large state-based healthcare system. A significant proportion of high-risk patients live large distances from tertiary level care. The greater accessibility of primary care services highlights the importance of supporting primary care physicians outside metropolitan areas to acquire or build the ability and capacity to care for children with CHD. Strengthening local primary and secondary services not only has the potential to improve the outcomes of high-risk patients, but also to reduce costs and burden associated with potentially avoidable travel from regional, rural, or remote areas to access specialist CHD services.

## Background

Paediatric cardiac care typically follows regionalised care models due to the high-complexity and acuity of the care provided. Higher volume centres are, in general, better able to provide this care with both theoretical and applied analyses indicating improved mortality and length of hospital admissions [1, 2]. The limitations of this model for organisation of care across large regions are the travel distance and timely access to appropriate care for some patients. Longer travel time for care has been shown to independently increase mortality and health care costs [3] and is particularly relevant in the Australian healthcare context. Global mapping of accessibility demonstrates Australia’s geographical challenges in providing high-quality healthcare resources outside of the cities with a tertiary-level paediatric cardiac centre [4].

In Queensland, the second-largest state in Australia, interventional cardiac care is provided at the Queensland Children’s Hospital (QCH), Brisbane. This city is located in the extreme south-eastern corner of a state with an area of 1.8 million km^2^. A significant number of Queensland’s paediatric patients are discharged to homes that are potentially thousands of kilometers from the surgical centre. This is important given the risk of morbidity in these patients, with recently published population data showing unplanned readmission rates for post-cardiac surgical children of 11% within 31 days of discharge [5]. Readmission morbidity is often significant, with median length of stay 5 days with an overall mortality of 1.8% for those readmitted and 6.25% for those requiring re-operation. From a long-term perspective, a recent report demonstrated 94% of CHD carers feel worried about their child and attend a higher number of consultations with both cardiologists (median 4/yr) and General Practitioners (GPs) (median 9/yr) compared to adult CHD patients [6]. Over one-third have also sought emergency care in the last 12 months [6].

There is a dearth of literature about the impact of distance from care on families with childhood heart disease. In other pediatric cohorts however, families that live long distances from tertiary centres encounter barriers such as lack of trust of local healthcare, financial stress, lower level of education and differing coping methods, including wishing problems away and blaming others [7]. Consequently, the organization of healthcare services to promote safe and effective delivery of high-acuity paediatric cardiac care and longer-term follow-up requires significant consideration for geographically dispersed populations, particularly for children with heart disease who may benefit from accessing appropriate care close to home [8].

Primary care physicians may be well placed geographically to provide timely accessible care to children with CHD. Prior research has indicated well-coordinated services developed in partnership with primary care may better meet families’ needs, increase continuity of care, reduce health system burden and be a cost-effective method of service delivery [8, 9]. Consequently, adequately supported primary care providers are a promising potential alternative to remote clinical monitoring and follow-up care requiring travel to tertiary facilities for care of children with CHD residing outside of urban centres where the specialist cardiac services are located.

To help inform future policy and practice recommendations, this study aimed to (i) describe the geographic distribution of patients accessing an Australian state-wide specialist paediatric CHD service and (ii) estimate travel time for accessing healthcare via general practitioners (primary), nearest paediatric centre (secondary) and specialist paediatric cardiac centre (tertiary) for families who did and did not live in the urbanised region where tertiary services are located.

## Methods

### Design

This study comprised a cohort of children (0–18 year) who accessed statewide specialist CHD services over a 3-year period (2019–2021 calendar years). The study involved a retrospective chart review for relevant data points, including age at intervention, type of intervention, cardiac diagnosis, place of residence, address of GP and address of closest secondary paediatric care facility.

### Defining the clinical sample

The Queensland Paediatric Cardiac Service performs approximately 400 surgical and 300 catheter-based procedures each year [10]. Patients were deemed to be at a relatively high risk and requiring follow up if at the point of discharge from inpatient care they had received cardiac surgery or catheter-based intervention during their admission at any stage within the three-year period. Locations for neighborhood of residence and general practitioner were collected from a centralized cardiac patient database at the Queensland Children’s Hospital where all paediatric cardiac interventions in the state are performed. A small number of patients from outside of the state, most commonly northern New South Wales, also received treatment at this tertiary centre. Only patients with a known current address were included, with a small number of patients with undocumented residences. Patient diagnosis and type of intervention were also collected for sub-group analysis based on relative risks based on cardiac lesion complexity.

### Defining geographical regions

Public health services in Queensland are provided through 16 Hospital and Health Services (HHS) [11]. These are statutory bodies, each governed by a Hospital and Health Board. Each HHS involves at least 1 referral hospital with either a hospital-based paediatric service, or a visiting medical officer model. Residential neighborhood data for patients was mapped to HHS boundaries to determine how many patients originated from each HHS region. Only children with residential addresses within Queensland were included in this analysis. While the most heavily urbanized region of Queensland, known as ‘South East Queensland’, is not defined legislatively, it is typically considered to include the 6 HHSs of Metro North, Metro South, Children’s Health Queensland, Gold Coast, Sunshine Coast and West Moreton. Collectively these HHSs are located in the extreme south-east corner of the state and account for an estimated 3.8 million out of the state’s 5.1 million residents [12], including the capital city of Brisbane, and the state’s tertiary cardiac referral hospital.

### Geographic information system mapping and analysis

Geographic Information Systems (GIS) software (ArcGIS online, [13]) was used to map the locations of the patient’s neighborhood of residence, primary care, secondary care, and tertiary care services. Calculations of distance and travel times were performed using exact address fields to estimate how long (time) and how far (distance in kilometers) each patient had to travel to access primary, secondary and tertiary level care. These calculations were performed by using a Google Maps Application Programming Interface (API) within Microsoft Excel Visual Basic and expressed as ‘drive time’ by road. This information was tabulated as percentages and numbers, medians and interquartile ranges.

## Results

### Patient characteristics

**Fig. 1:**
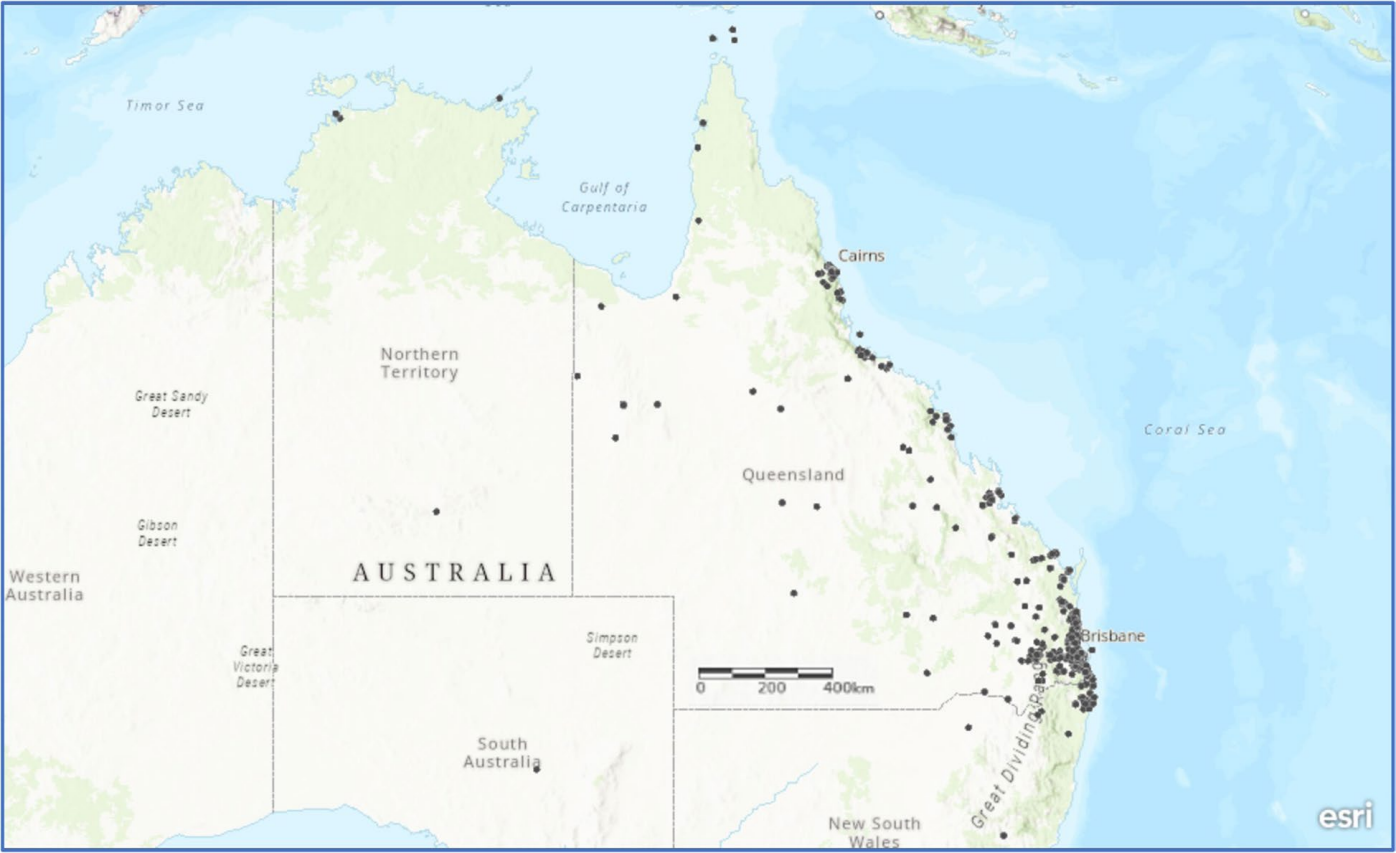
Residential location of patients after intervention at Queensland Children’s Hospital (includes patients from out-of-state)

 A total of 1019 individual patients underwent cardiac intervention or had a new diagnosis of rheumatic heart disease (RHD), with a total of 1137 procedures overall (Table 1). Sixty-two patients were from outside of Queensland and were excluded from analyses. For the remaining 957 patients, residential locations were greatly dispersed across the state and 289 (30.2%) lived outside of the urbanised South-East Queensland (Fig. 1) region. The median (and interquartile) age was similar for those residing outside (3.6years (IQR 0.4 to 10.8 years)) and within South East Queensland (3.8years (IQR 0.2 to 10.8 years)). For those living within South East Queensland 38 patients (5.6%) had either a single ventricle or 1.5 ventricle circulation, compared to 22 patients (7.6%) having a single ventricle circulation while living outside South East Queensland. Interventional catheterisation was performed for 315 patients (31%), and of those, electrophysiology procedures were performed in 175. Cardiac surgery was performed in 703 (68.9%) patients. Patient and intervention characteristics were similar when comparing those who reside close to the tertiary centre and those more remote, except for those with rheumatic heart disease, with a greater prevalence of repaired RHD living outside SEQ.

**Table 1:**
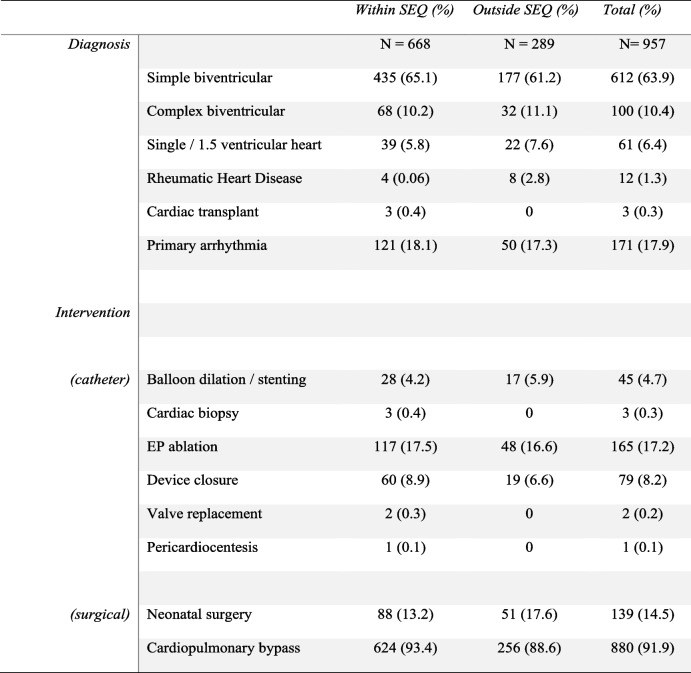
Patient characteristics

*EP* Electrophysiology, *SEQ *South East Queensland

### Access to care

**Fig. 2:**
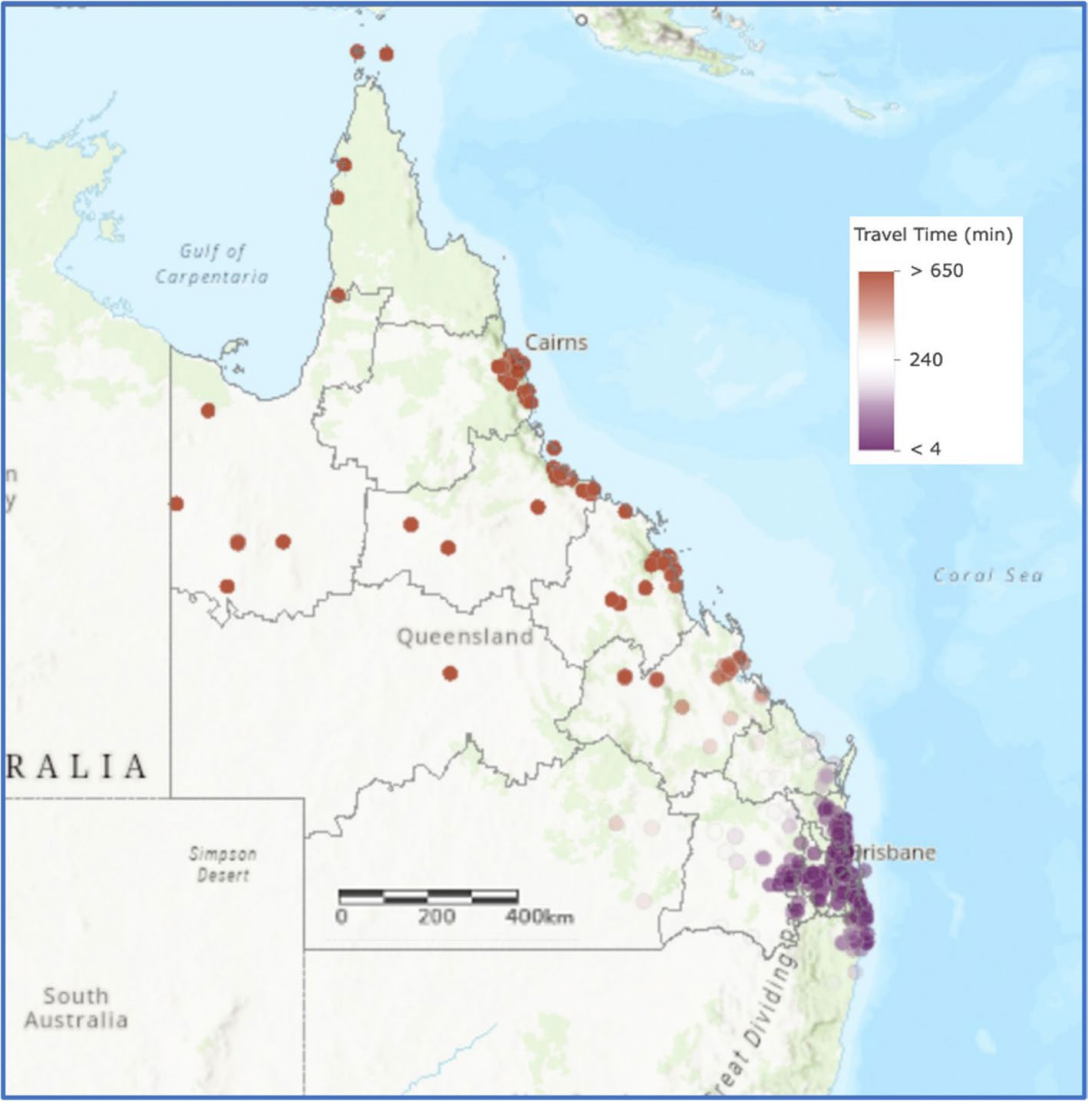
Travel time of patients from residential address to tertiary level care

 A visualization of travel time for patients to access tertiary level care is presented in Fig. 2. Median distance to QCH for those in SEQ was 33.4 km, and 953.3 km if outside SEQ (Table 2). Calculated travel time reflected this variability in distance. While the median travel time for those in SEQ was 36.3 min, this became more than 11 h for those beyond this region (Table 3). The longest recorded travel time for a patient in South East Queensland was 2.9 h compared to 28.6 h for a patient living in far North Queensland. Both travel time and travel distance to tertiary care were even higher for RHD patients outside SEQ than for the total cohort outside SEQ (15 h 12 m, 1345 km respectively).

**Table 2:**
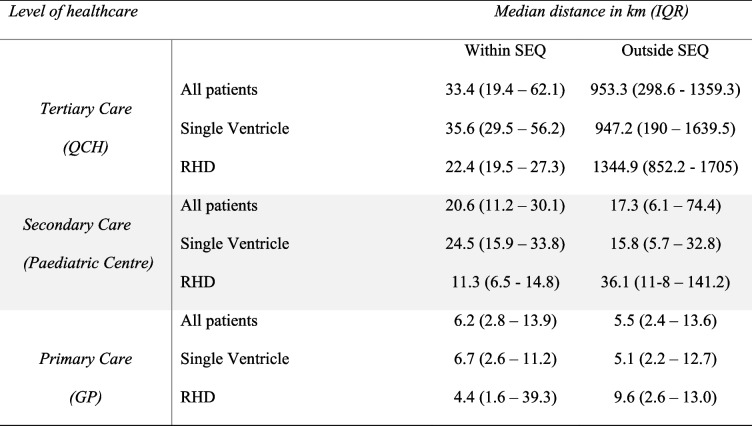
Distance of travel from patients residential address to care

*GP* General practitioner, *IQR *Interquartile range, *QCH *Queensland Children’s Hospital, *RHD *Rheumatic heart disease, *SEQ *South East Queensland

**Table 3:**
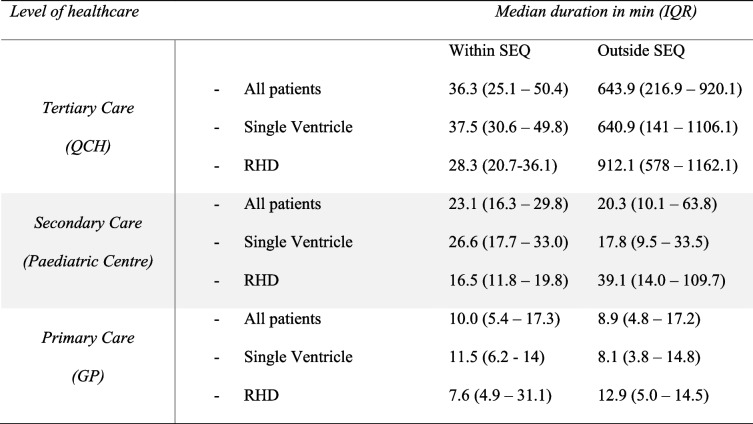
Travel time from patients residential address to care

*GP* General practitioner, *IQR *Interquartile range, *QCH *Queensland Children’s Hospital, *RHD *Rheumatic heart disease, *SEQ *South East Queensland

**Fig. 3:**
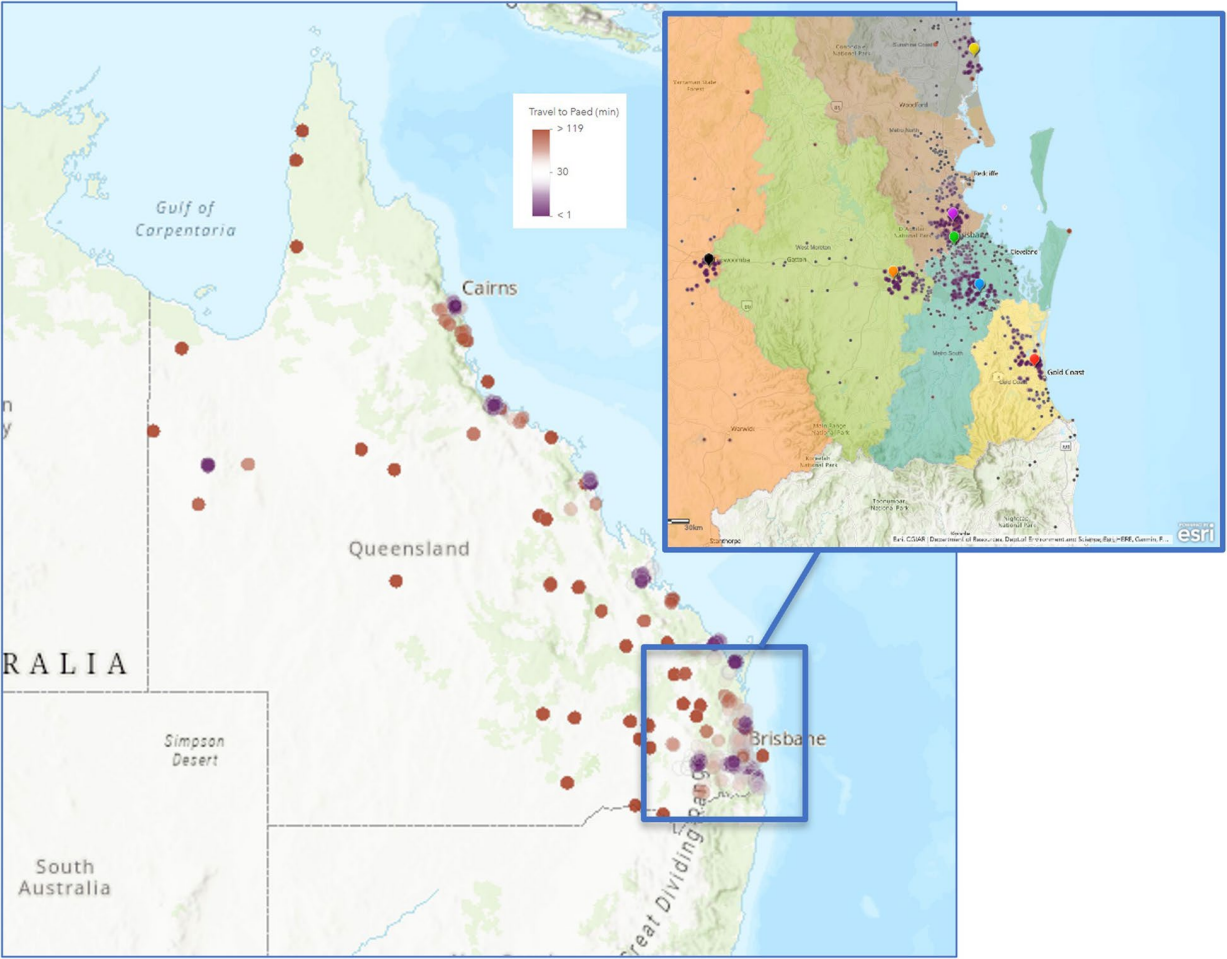
Travel time of patients from residential address to secondary level care at a paediatric centre. (Inset: Secondary Centres within South East Queensland. Coloured areas represent each HHS, coloured dots represent each secondary centre)

 Distance to access acute paediatric (secondary level) care was comparatively closer due to the presence of paediatric hospital-based services in metropolitan centres dispersed across the state (Fig. 3). Median distance to secondary level pediatric care for those in SEQ was 20.6 km, and 17.3 km if outside SEQ (with a mean distance outside SEQ of 68.8 km). Median travel time if living outside SEQ was 20.3 min (with a mean time of 55.8 min).

**Fig. 4:**
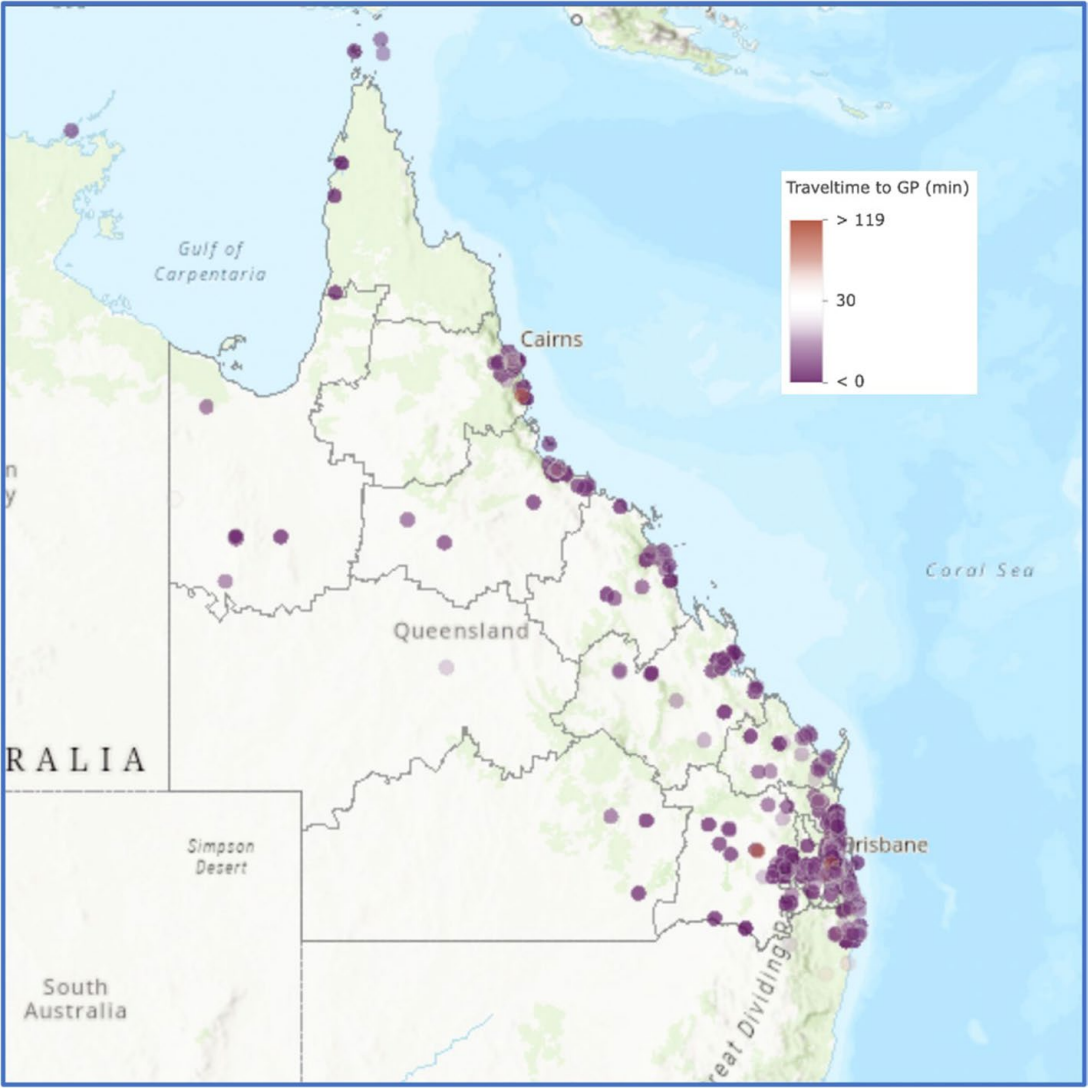
Travel time of patients from residential address to primary level care (GP)

 Access to primary level care via General Practitioners (GPs) was both closer to home and similar for patients regardless of residential location (Fig. 4). Median travel time to the GP in SEQ was 10.0 min, and 8.9 min outside SEQ (Tables 2 and 3). RHD patient travel time and distance to their primary care physician was 12 min and 9.6 km.

## Discussion

This study represents the first application of GIS mapping to paediatric cardiac care in Australia and described the extent to which distance is a key challenge for accessing care for many patients with childhood heart disease and their families. It also highlights that distance is a significant hurdle in addressing the barriers to improved Indigenous care for those in the north of the country. Collaborative work between adult cardiac clinicians and geographers in Australia has previously indexed the relative inequity of providing acute cardiac care to the adult population in Australia [14]. Our analysis extended this work by demonstrating the significant variability in access to higher levels of care for our paediatric cardiac patients across the state. Importantly, our analysis highlighted potential opportunities for improving access to care for pediatric patients by utilising primary care providers and secondary level hospital sites. It is also of note that children with Rheumatic Heart Disease in our study are much more likely to live outside the metropolitan area and have less accessible tertiary care. This is important, given that young patients with RHD are usually of Indigenous status and have a high rate of death and non-fatal complications from their disease (up to one-fifth of uncomplicated cases in a recent report by Stacey and colleagues [15]) with complication risk highest in the first 6 months after diagnosis.

These findings raise important issues regarding equity of access to care for children with CHD in Queensland. Systemic differences in access across regions, such as that observed in the present study, are problematic for achieving equitable care, a key domain of health care quality [16]. It has been shown, for example, that poor travel time access reduces the utilisation of healthcare facilities [17]. There are now proposed frameworks to address spatial inequity in health delivery. While this study addresses the *accessibility* component of spatial inequity, other equally important domains of *availability*, *affordability*, *accommodation*, and *acceptability* remain to be assessed in our context [18].

The geographic limitations of care access in Queensland need to be contextualised to existing research which demonstrates improved outcomes when paediatric care is centralized [1]. Much of this work has been done in the United States where the majority of congenital heart surgery centres lie within 40 km of another [1]. This is in comparison with Queensland where patients travel an average of 308 km. Median travel distance to US centres has been reported at 62 km, markedly lower than the median travel distance of 953 km for Queensland patients outside of South East Queensland.

There is no data on care centralisation for CHD patients in the Australian setting, and it is unclear if the inequities in access observed in our mapping negate the beneficial outcomes observed in other countries. Reassuringly, overall survival in Australian cardiac surgical patients compares favourably with international data from recent registry analysis [19], however this does not include an analysis of cost or patient experience. In the absence of any feasible option of providing tertiary level care across more centres, other care models need to be considered with a balance between centrally and locally delivered care. Based on the data from our gap analysis GPs are well placed geographically, to be included in any revised model of care. Any redistribution of care services to a local setting will have additional cost reduction to the health care system in transportation savings and reduced loss of income to families.

Existing literature may point to the feasibility and effectiveness of involving primary care providers in the care of children with CHD. While studies are limited, emergent research in the field, in non-Australian settings, has indicated GPs are both comfortable in providing ‘shared-care’ to children with CHD [20] and families are more likely to access their GP for most health queries [21, 22]. However, there is evidence that the perceived role of the primary care physician can be very different between parents and physicians [23] and other data suggesting that communication between the tertiary hospital and local physicians needs to be improved at the point of discharge [24]. GPs may therefore be well placed to contribute as key stakeholders in an improved model of care provision across a state as vast as Queensland. This assumption however requires additional research regarding the role of the primary care physician in Australia, feasibility of a model with increased reliance on primary care contributions, and examination of the extent to which these findings generalize to other regions.

### Limitations

A limitation of this study was the output received through the Google Maps API script. This produces a drive time calculation and does not take into account faster journeys via air or other important considerations such as public transport, car ownership, or variability in different traffic conditions. It also cannot take into consideration shorter travel times for acute retrieval of patients with the Royal Flying Doctor Service. Nonetheless, despite these limitations drive time was a meaningful indicator of the overall time taken for remote patients to access tertiary care, and while air travel may be faster for some patients, our assessment was focused on access to care for patients at risk of deterioration in clinical condition. Such patients requiring care would not be eligible for commercial air travel and would have to wait for aeromedical retrieval. A further limitation is the unknown burden of unplanned readmission in our cohort. Further local analysis will be required to compare our readmission burden to that published overseas.

## Conclusion

A geographic analysis of the provision of different levels of care to children with CHD in Queensland demonstrates the problem of distance in our attempts to optimize care to our patient cohort. While a large percentage of patients live significant distances from the surgical centre in Brisbane, primary care physicians are geographically well placed to contribute to improved models of care. The greater accessibility of primary care services highlights the importance of supporting primary care physicians outside metropolitan areas. A well-supported, knowledgeable primary care physician has the potential to improve the outcomes of high-risk patients, including those in our Indigenous communities, but also to reduce costs and burden associated with potentially avoidable travel from regional, rural, or remote areas to access specialist CHD services.

## Data Availability

The datasets used and/or analysed during the current study are available from the corresponding author on reasonable request.
